# Stable habituation deficits in the early stage of psychosis: a 2-year follow-up study

**DOI:** 10.1038/s41398-020-01167-9

**Published:** 2021-01-05

**Authors:** Suzanne N. Avery, Maureen McHugo, Kristan Armstrong, Jennifer Urbano Blackford, Neil D. Woodward, Stephan Heckers

**Affiliations:** 1 Department of Psychiatry and Behavioral Sciences, Vanderbilt University Medical Center, Nashville, TN USA; 2 Research Health Scientist, Research and Development, Department of Veterans Affairs Medical Center, Nashville, TN USA

**Keywords:** Hippocampus, Schizophrenia

## Abstract

Neural habituation, the decrease in brain response to repeated stimuli, is a fundamental, highly conserved mechanism that acts as an essential filter for our complex sensory environment. Convergent evidence indicates neural habituation is disrupted in both early and chronic stages of schizophrenia, with deficits co-occurring in brain regions that show inhibitory dysfunction. As inhibitory deficits have been proposed to contribute to the onset and progression of illness, habituation may be an important treatment target. However, a crucial first step is clarifying whether habituation deficits progress with illness. In the present study, we measured neural habituation in 138 participants (70 early psychosis patients (<2 years of illness), 68 healthy controls), with 108 participants assessed longitudinally at both baseline and 2-year follow-up. At follow-up, all early psychosis patients met criteria for a schizophrenia spectrum disorder (i.e., schizophreniform disorder, schizophrenia, schizoaffective disorder). Habituation slopes (i.e., rate of fMRI signal change) to repeated images were computed for the anterior hippocampus, occipital cortex, and the fusiform face area. Habituation slopes were entered into a linear mixed model to test for effects of group and time by region. We found that early psychosis patients showed habituation deficits relative to healthy control participants across brain regions, and that these deficits were maintained, but did not worsen, over two years. These results suggest a stable period of habituation deficits in the early stage of schizophrenia.

## Introduction

Habituation, defined as the decrease in response to repeated stimulus exposures, is an elementary and ubiquitous form of behavioral plasticity^1^. Acting as a sensory ‘firewall’^2^, habituation is thought to gate the vast amounts of irrelevant and repeated sensory information experienced in our everyday environment, allowing prioritization of salient and actionable items. Rapid habituation may conserve limited processing resources and act as a building block for normal cognitive function^3,4^. In contrast, failure to rapidly habituate has been proposed to lead to diminished attentional focus^5^, cognitive fragmentation^6^, and inappropriate behavioral responses^7^. Thus, habituation may be a foundation for cognitive integrity in schizophrenia wherein even modest impairments in habituation may be capable of producing clinical impairment^6^.

Impairments in habituation and sensory filtering in schizophrenia have been recognized for over a century^8^ and are now considered core features of the illness^7,9^. Habituation deficits have been consistently identified at all levels of measurement, including at the behavioral, electrophysiological, and neural level. Historically, habituation deficits in schizophrenia were identified using well-validated measurements such as habituation of eye-blink startle^9,10^ and autonomic nervous system response^11,12^. More recently, studies using electroencephalogram (EEG) and functional magnetic resonance imaging (fMRI) measures have demonstrated neural habituation deficits in schizophrenia, including reduced habituation to auditory evoked potentials^13–15^, and reduced habituation to repeated visual stimuli^16–18^. Although the specific neural processes underlying habituation deficits remain elusive^19^, at all levels of measurement, habituation is thought to reflect inhibitory mechanisms of plasticity^20^. Studies in *Drosophila* have shown that habituation requires effective inhibition of excitatory response via a centralized, network-level potentiation of inhibitory synapses^21,22^. Inhibitory function is disrupted in schizophrenia, and failure of inhibition is among the most compelling explanations for the widely-observed sensory processing impairment seen in patients^23^.

An important brain region associated with inhibitory deficits in schizophrenia is the hippocampus^24–26^. Playing a fundamental role in filtering of incoming sensory information in support of learning, the hippocampus rapidly habituates to repeated visual information^27–31^ in healthy individuals. However, schizophrenia patients show deficits in hippocampal habituation^16–18^ and inhibitory function^24^ to repeated visual stimuli, with inhibitory deficits linked to impaired information processing^32^. Recent findings convincingly show inhibitory deficits also exist in the visual cortex^33,34^ in schizophrenia. Repeated visual stimuli elicit robust habituation in the visual cortex^35^ and fusiform gyrus^35,36^ in healthy individuals, while schizophrenia patients show habituation deficits in both regions^16,17^. Together, these findings suggest correspondence between habituation and inhibitory function in the hippocampus and ventral visual stream, in-line with evidence in the non-human literature that implicates habituation as a deficit of inhibition.

Disruption of inhibitory function may be involved in the pathogenesis of schizophrenia^37^. In recent years, there has been growing interest in defining neural mechanisms that could be used as treatment targets for interventions that may attenuate progression of illness. The early phase of psychotic illness has been proposed as a critical period during which deterioration progresses rapidly and interventions may be most impactful^38^. Habituation, first described in *Aplysia*^39^, is a fundamental process that is highly conserved across species, making it an ideal mechanism for translational study. However, understanding whether habituation deficits progress with illness is a crucial first step. Although neural habituation deficits have been observed in both early^17^ and chronic stages of schizophrenia^16,18^, differences in task administration and analysis techniques across studies have made it difficult to determine whether habituation deficits differ by illness stage.

We previously identified habituation deficits in three regions—the anterior hippocampus, occipital pole, and fusiform face area (FFA)—in a cohort of early psychosis patients with less than 2 years of illness^17^. The aim of the current study was to determine whether these deficits progressed over the next two years of illness. Participants who completed the cohort study were asked to return for a follow-up visit 2 years later. During the follow-up visit, participants repeated the visual-stimulus repetition task administered at baseline using an alternate image set. This is the first longitudinal analysis of the repetition task. We hypothesized that habituation deficits progress over two years of illness.

## Methods and material

### Participants

One-hundred thirty-eight participants (70 early psychosis patients and 68 healthy control participants) were enrolled in a prospective longitudinal study of habituation between May 2013 and November 2017 and followed for two years (Supplementary Figs. 1 and 2). Cross-sectional baseline data from some of our participants have already been reported^17^ (62 early psychosis patients and 68 healthy control participants); this is the first report of follow-up data.

All participants were assessed by a trained rater using the Structured Clinical Interview for the DSM-IV (SCID I-P)^40^ and diagnoses were confirmed by a senior psychiatrist (S.H.). Patients in the early stage of a psychotic disorder were recruited from the outpatient clinics and inpatient units of Vanderbilt Psychiatric Hospital. Inclusion criteria included: (1) age 13–40; (2) premorbid IQ above 75; (3) <2 years of psychotic illness; and (4) meeting criteria A for schizophrenia^40,41^. Early psychosis participants were excluded if they reported active substance use or dependence in the past month or if a psychotic disorder due to a medical condition was diagnosed. Additionally, only patients meeting criteria for a schizophrenia spectrum disorder (schizophreniform disorder, schizoaffective disorder, or schizophrenia) at 2-year follow-up were included in this analysis. Patients were assessed for current mood, psychotic symptom severity, and antipsychotic medication dose at baseline and two-year follow-up (see Supplementary Methods). More than 85% of the patient sample were treated with antipsychotic medication at the time of the study.

Healthy control participants were recruited from the community via advertisement. Healthy control participants were excluded if they met criteria for any Axis I disorder^40,41^ at enrollment and at the end of the study, had a first-degree relative with a psychotic illness, or had current psychotropic medication use. Groups were recruited with the goal to match for mean age, gender, race, and parental education (Table 1).

**Table 1:** Demographics.

Demographics	Healthy control, *n* = 68	Early psychosis, *n* = 70	Healthy control vs. early psychosis	
	Mean ± SD	Mean ± SD	Statistic	*p*
Age at enrollment, years	22 ± 2.9	21 ± 3.8	*F*_1,137_ = 0.22	0.64
Sex, % male	72%	77%	*Χ*^2^_1_ = 0.47	0.49
Race, white/black/other	53/11/4	53/16/1	*Χ*^2^_2_ = 2.70	0.26
Handedness, % right	91%	94%	*Χ*^2^_1_ = 0.50	0.48
Premorbid IQ, WTAR	112 ± 10.5	103 ± 16.0	*F*_1,129_ = 14.66^a^	<0.001*
Participant’s education, years	15 ± 1.9	13 ± 2.3	*F*_1,137_ = 10.79	<0.001*
Parental education, years	15 ± 2.4	15 ± 2.7	*F*_1,137_ = 0.36	0.55

Clinical characteristics	Baseline, *n* = 64		Follow-up, *n* = 59		Baseline vs. follow-up	
	Mean ± SD	Range	Mean ± SD	Range	Statistic	*p*
Diagnosis, SZF/SZ/BP	42/19/3	−	16/43/0	−	−	−
Duration of illness, months	7 ± 5.7	1–24	31 ± 5.5	24–48	−	−
CPZ, mg	271 ± 181.3	0–750	196 ± 219.7	0–900	F_1,122_ = 4.34	0.04*
Antipsychotic treatment, %	84%	−	64%	−	*Χ*^2^_1_ = 6.49	0.01*
PANSS Total	66 ± 20.1	34–114	51 ± 14.9	33–104	F_1,122_ = 22.52	<0.001*
PANSS-Positive	17 ± 6.9	7–36	13 ± 4.6	7–27	F_1,122_ = 13.40	<0.001*
PANSS-Negative	17 ± 7.8	7–37	12 ± 6.3	7–32	F_1,122_ = 14.83	<0.001*
PANSS-General	33 ± 9.1	17–59	26 ± 7.0	17–46	F_1,122_ = 18.77	<0.001*
HAM-D	9 ± 6.3	0–24	5 ± 4.2	0–15	F_1,122_ = 14.94	<0.001*
YMRS	3 ± 4.4	0–20	2 ± 2.7	0–15	F_1,121_ = 1.18^b^	0.28

*WTAR* Wechsler Test of Adult Reading, *SZF* schizophreniform, *SZ* schizophrenia, *BP* bipolar disorder with psychotic features, *CPZ* chlorpromazine equivalent, *PANSS* Positive and Negative Syndrome Scale, *HAM-D* Hamilton Depression Rating Scale—17 item, *YMRS* Young Mania Rating Scale. Asterisk (*) denotes significant *p* values (*p* ≤ 0.05).

^a^WTAR scores are reported for native English speakers (early psychosis, *n* = 68; healthy control, *n* = 63).

^b^One patient missing YMRS at baseline.

All participants provided written informed consent and received monetary compensation for their time. The study was approved by the Vanderbilt University Institutional Review Board, Nashville, TN.

### Clinical assessment and measures

We collected clinical data at enrollment and two-year follow-up to characterize trajectory of illness. The full clinical assessment has been previously detailed^42^. Briefly, early psychosis participants completed in-person clinical interviews, including the SCID^43^, Positive and Negative Syndrome Scale (PANSS)^44^, Depression Rating Scale (HAM-D)^45^, Young Mania Rating Scale (YMRS)^46^, and Screen for Cognitive Impairment in Psychiatry (SCIP)^47^. Duration of psychosis was calculated as the date of onset of psychosis until the date of study enrollment. All data gathered during the in-person interviews were augmented by extensive review of all available medical records. Final diagnoses were made by psychiatrist S.H. during diagnostic consensus meetings.

### Experimental paradigm

#### Repetition task

Participants completed a repetition task designed to elicit habituation to repeated stimuli^16,17^ (Supplementary Fig. 3). The repetition task included four 2-min runs of a repeated neutral face (run 1 and 2) and a repeated neutral object (table, box of tissues; run 3 and 4). Images were presented for 500 ms followed by a 500 ms black screen. To promote and assess attention, a target detection task was included. Targets were small versions of the face or object images (25% of original size^36^) presented on 10% of trials. No targets were presented during the first 10 s of stimulus repetitions. Participants were asked to press a button during each small image presentation. Target detection was high (detection means > 93%) and similar between groups and across visits (*p*s > 0.36; see Supplementary Results).

### Imaging data acquisition and processing

Imaging data were collected on a 3 T Philips Intera Achieva magnetic resonance imaging (MRI) scanner located in the Vanderbilt University Institute for Imaging Science. A high-resolution T1-weighted fast field echo (FFE) structural scan and four 2.5-min functional echo planar images (EPI) were acquired for each subject (see Supplementary Methods for details). Functional data were motion corrected, coregistered to the subject’s structural image, and smoothed using a 6 mm Gaussian kernel in SPM12 (http://www.fil.ion.ucl.ac.uk/spm). The first-level (participant) temporal model was estimated on smoothed, native-space functional data using a general linear model^48^. Small images, outliers, and motion (rotation, translation, relative displacement; see *Quality control* below) were entered into the first-level general linear model^48^ as regressors of no interest and residuals were written for second-level analysis. No temporal filter was applied because slow signal changes may represent habituation to stimulus category.

#### Quality control

Functional images were visually inspected for artifacts and signal dropout. Motion (translation, rotation, and relative displacement) and outliers were computed using Artifact Detection Tools (ART; Neuroimaging Informatics Tools and Resources Clearinghouse (NITRC)). Outlier volumes were defined as volumes with global intensity change > 5 SD from mean, or motion > 3 mm. Functional data with ≥2 outlier volumes within the 10 s habituation window (see *Habituation* below) were excluded. One patient had 2 outlier volumes at follow-up and was removed from follow-up analysis. The majority of participants had no outliers at either study visit (baseline, 0%; follow-up, 1%; *ps* ≥ 0.41). Motion was low and similar between groups (*ps* ≥ 0.16; Supplementary Table 1).

### Data analysis

#### Regions of interest (ROIs)

Our goal was to test whether previously identified habituation deficits^17^ in the anterior hippocampus, occipital pole, and fusiform face area (FFA) were maintained over 2-year follow-up. Therefore, ROIs were delineated the same way as in our prior cohort analysis^17^. Subject-specific hippocampal and occipital pole masks were created using in-house automated multiatlas segmentation techniques^49,50^. Separate baseline and follow-up ROIs were created to account for potential changes in gray matter volume. Hippocampal masks were manually segmented at the uncal apex^51,52^ by a trained rater (S.A.) to create anterior hippocampal ROIs. The FFA was defined in each subject using a localizer task^53^ (Supplementary Methods). Although the FFA was originally proposed as a face-selective region, its role has since been understood as one involving visual expertise^54,55^. Because our stimuli were common objects (table, box of tissues) we expected FFA involvement in processing. Three healthy control participants (2 at baseline, 1 at follow-up) and six patients (all at follow-up) were excluded from FFA analyses because: the localizer scan was not collected due to scanning time constraints (healthy control = 2, early psychosis = 4); or no FFA clusters were identified (healthy control = 1, early psychosis = 2).

#### Habituation

Habituation was defined as the slope of change in neural signal over repeated stimulus presentations. Consistent with our prior cohort analysis^17^, the habituation window was defined as the window between novelty response and the return to baseline in the healthy control group. Novelty response was defined as the peak signal magnitude following the initial presentation of a stimulus. To minimize confounding effects of repeated exposure to the scanner environment, the habituation window was calculated from the baseline visit. Healthy control signal peaked 8 s following stimulus onset and returned to baseline by 18 s following stimulus onset, resulting in a 10 s habituation window. This window is consistent with prior fMRI studies of habituation in healthy participants^56^; additionally, this window did not include any small image presentations to minimize the effects of small images on habituation results^57^. Novelty response was defined as the fMRI signal amplitude at the beginning of the habituation window. As our prior cohort analysis^17^ revealed between-group differences in habituation to objects, we analyzed the repeated objects run here. For consistency with prior analyses^16,17^ the first objects run was used for habituation analyses, as neural response is minimal following initial stimulus exposure.

Average timeseries for each ROI was extracted from participants’ residual time course data using MarsBar^58^. Residual time courses were plotted by hemisphere for each subject and calculations were conducted using Matlab R2017b (The MathWorks Inc., Natick, MA). Signal in the left and right hemispheres were highly correlated across regions; to increase statistical power and minimize type I error, data were averaged across hemispheres.

#### Habituation slope

**Fig. 1:**
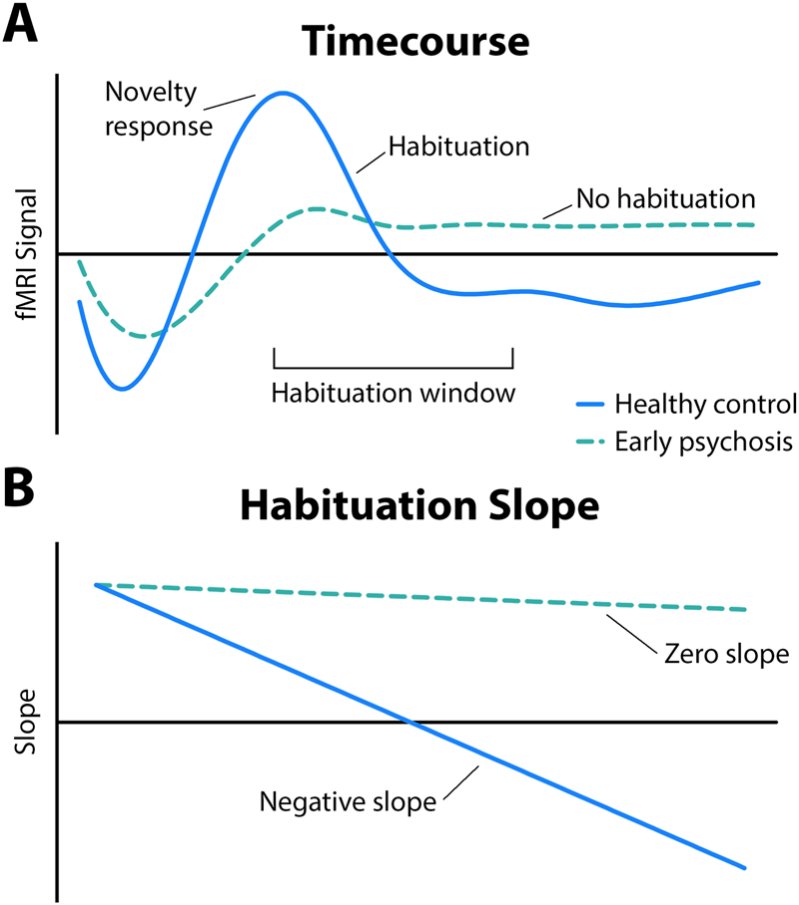
We studied habituation of neural response over time. Functional magnetic resonance imaging (fMRI) signal was extracted for a 10 s window beginning with the novelty response (**A**) and habituation slopes were calculated (**B**). Habituation was characterized as the change in fMRI signal over time with more negative slopes indicating greater habituation.

Habituation is highly dependent on the magnitude of the novelty response—that is, there is more opportunity for signal to attenuate over time if signal is initially high. Because we were interested in examining differences in rate of habituation independent of differences in novelty response, we calculated a normalized habituation slope (*b*′), corrected for novelty response differences, for each participant^59–61^. Habituation slope (*b*′) values were calculated for each participant using linear regression analysis (see Supplementary Methods for details). Fig. 1 summarizes the habituation slope analysis.

### Statistical analysis

Linear mixed effects models tested for habituation rate differences by group and time, with group included as a fixed factor, time as a repeated factor, and participant as a random factor. Because habituation deficits within each region were previously identified in an overlapping sample^17^, regions were tested separately for time and group effects. Follow-up within-group one-sample *t*-tests tested whether habituation slopes were significantly less than zero (h0 = 0, no habituation; *p* ≤ 0.05). Cohen’s *d* effect sizes were calculated using the formula contained in ref. ^62^. Before completing the study, we conducting a power analysis and determined that with the present sample size, we would have 80% power to detect an effect size of *d* = 0.47 in our primary longitudinal analysis. Spearman correlations tested for associations between habituation and cognitive and cognitive measures. Spearman correlations test for monotonic rather than linear relationships between variables. Spearman correlations were corrected for multiple comparisons across measures within group, time, and region (FWE-adjusted *p* ≤ 0.05). Statistical analyses were performed using SAS software v9.4 (SAS Institute Inc., Cary, NC).

## Results

### Clinical sample

The patient sample was young (mean age 21 years), primarily male (77%), Caucasian (76%), and educated (13 years education; Table 1). The majority (47, 67%) were diagnosed with schizophrenia or schizoaffective disorder after two years of follow-up. Within this group, 17 already met criteria at study entry, 26 progressed from an initial diagnosis of schizophreniform disorder, and 4 converted from an initial diagnosis of psychotic bipolar disorder. A minority (17, 24%) were diagnosed with schizophreniform disorder at study entry and did not progress to another diagnosis within two years. Early psychosis patients had lower clinical symptoms and CPZ equivalent dose at follow-up than at baseline (Table 1).

### Exclusions and attrition

Of the 138 participants, data at either baseline or follow-up for 16 participants were excluded due to: low data quality at baseline (six patients, one healthy control) or follow-up (three patients, three healthy controls; see Quality control); or an ineligible diagnosis at follow-up (two patients, one healthy control; see Supplementary Methods for full details). Additionally, 6 early psychosis patients and 12 healthy control participants were unavailable to return for the follow-up visit. This resulted in an analysis group of 104 longitudinal participants (53 patients, 51 healthy controls), and an additional 34 participants with baseline (11 patients, 16 healthy controls) or follow-up data (6 patients, 1 healthy control; Supplementary Figs. 1 and 2). The 6 early psychosis participants without a 2-year follow-up visit had been diagnosed with schizophrenia (*n* = 2) or schizophreniform disorder (*n* = 4) at study entry and were not demographically and clinically different from early psychosis participants who completed both visits (*p*’s ≥ 0.53).

### Novelty response

Novelty responses were similar between groups (Supplementary Table 2) and were significantly greater than baseline across regions (Supplementary Table 3).

### Habituation

**Fig. 2:**
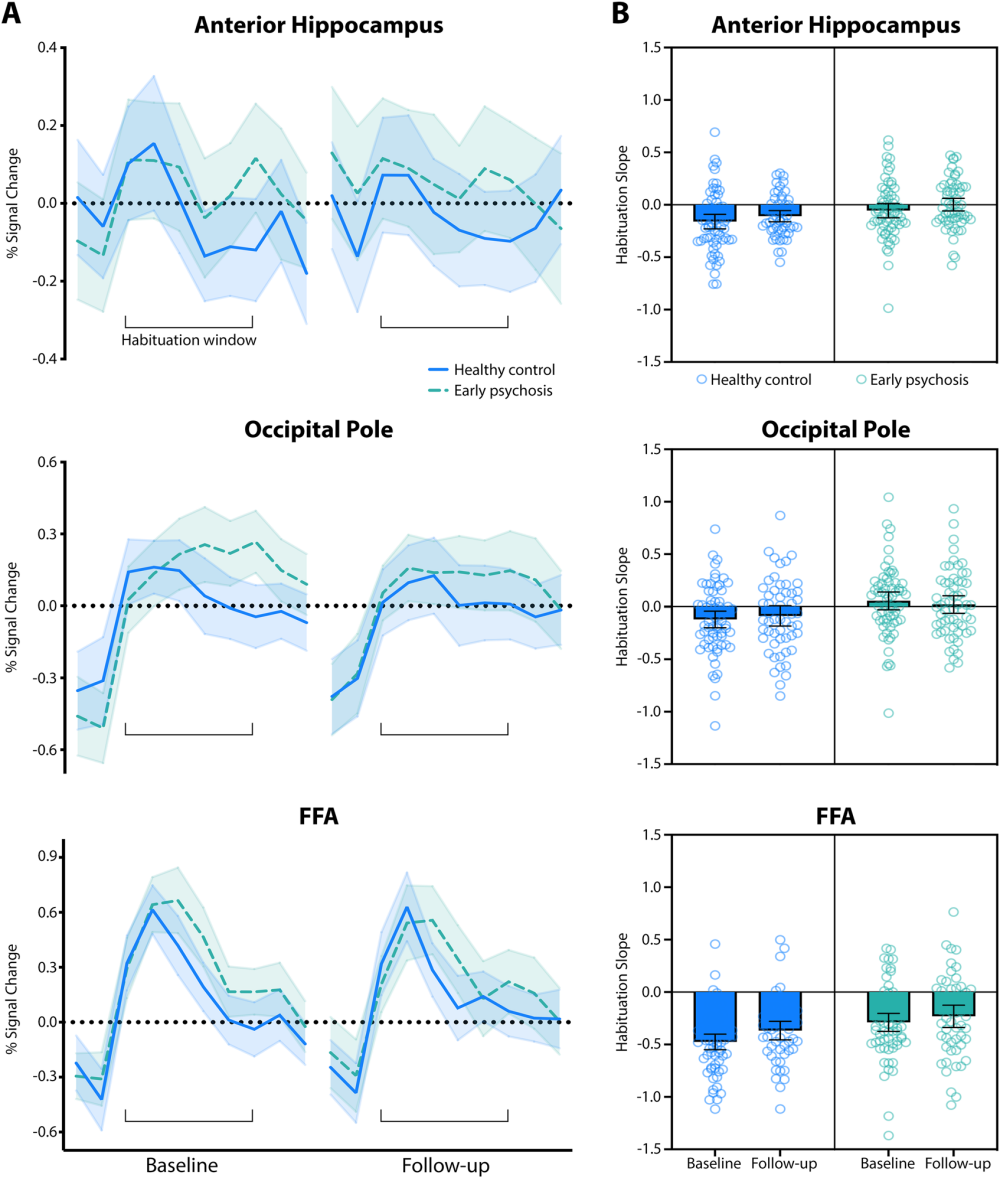
Neural habituation to repeated objects at baseline and 2-year follow-up by group. Mean fMRI signal for the first 20 s of the repeated objects task is shown for each visit by ROI (**A**). Habituation slopes were calculated over a 10 s habituation window, with more negative slopes indicating greater habituation. The healthy control group showed negative habituation slopes across ROIs (**B**). The early psychosis group showed negative habituation slopes in only the FFA, and habituation slopes in all ROIs were smaller than in the healthy control group. Habituation slopes in both groups were consistent over time. Bars show the mean habituation slope by group (**B**); shaded areas (**A**); and error bars (**B**) indicate the 95% confidence interval.

Habituation slopes at baseline and 2-year follow-up are presented in Fig. 2. We found a main effect of group in each region (anterior hippocampus, *F*_1,136_ = 11.92, β = 0.11, SE = 0.03, effect size = 0.57, *p* = 0.001; occipital pole, *F*_1,136_ = 9.91, β = 0.14, SE = 0.05, effect size = 0.51, *p* = 0.002; FFA, *F*_1,136_ = 12.52, β = 0.16, SE = 0.05, effect size = 0.58, *p* = 0.001), with early psychosis patients habituating ~11–16% slower than healthy control participants across regions (anterior hippocampus = 11%, occipital pole = 14%, FFA = 16%). The FFA showed a trend effect of time (*F*_1,93_ = 3.78, β = −0.08, SE = 0.04, effect size = 0.35, *p* = 0.055), with less habituation in both groups at follow-up compared to baseline (no group by time interaction, *p* = 0.58). Neither the anterior hippocampus nor the occipital pole showed a main effect of time (*p*s ≥ 0.11) or a group by time interaction (*p*s ≥ 0.45), indicating habituation rates remained stable over time in both groups. Habituation rates did not differ between patients with a follow-up diagnosis of schizophrenia vs. schizophreniform disorder (*p*s ≥ 0.55). Group results were similar when examining habituation slopes uncorrected for novelty response (Supplementary Table 4).

We conducted follow-up one-sample *t*-tests to further characterize habituation within groups (Table 2). As expected, there was evidence of significant habituation in healthy control participants at both baseline and follow-up in all regions. In contrast, early psychosis patients showed evidence of habituation at both baseline and follow-up in only one region, the FFA. Although there was a trend for anterior hippocampal habituation at baseline in the early psychosis group (*p* = 0.06), there was no evidence of habituation at follow-up. There was no evidence of occipital pole habituation at either timepoint in early psychosis. Together, these findings suggest that habituation is significantly attenuated in the FFA and deficient in the anterior hippocampus and occipital pole in early psychosis compared to healthy controls, and these impairments are maintained over 2 years of illness.

**Table 2:** Habituation.

Brain Region	Baseline				Follow-up				
	M ± SD	*t*	*df*	*p*	M ± SD		*t*	*df*	*p*
Healthy control									
Anterior hippocampus	−0.16 ± 0.3	−4.63	66	<0.001*	−0.11 ± 0.2		−3.97	51	<0.001*
Occipital pole	−0.12 ± 0.3	−3.02	66	0.002*	−0.09 ± 0.4		−1.73	51	0.04*
FFA	−0.48 ± 0.3	−12.82	64	<0.001*	−0.37 ± 0.3		−8.33	50	<0.001*
Early psychosis									
Anterior hippocampus	−0.06 ± 0.3	−1.62	63	0.06	0.00 ± 0.2		0.09	58	0.54
Occipital pole	0.06 ± 0.3	1.33	63	0.91	0.02 ± 0.3		0.56	58	0.71
FFA	−0.29 ± 0.3	−6.71	63	<0.001*	−0.23 ± 0.4		−4.36	52	<0.001*

Mean values ± standard deviations are shown for each group. Asterisk (*) denotes significant *p* values (*p* ≤ 0.05).

*M* mean, *SD* standard deviation, *FFA* fusiform face area.

### Clinical and cognitive correlates of habituation

To determine whether individual variability in habituation rate was associated with function, we conducted correlations with clinical and cognitive measures. Habituation rates were not associated with general measures of cognition (SCIP) or verbal IQ (WTAR) in either group (Supplementary Table 5). There was a negative correlation between negative symptoms and habituation in the anterior hippocampus at follow-up (*r* = −0.38, FWE-adjusted *p* = 0.03). Negative symptoms were not correlated with baseline habituation in any region. Other clinical measures of psychosis, mood, duration of illness, and CPZ equivalent dose were not correlated with habituation at either visit.

## Discussion

Our findings provide evidence for persistent habituation deficits in schizophrenia spectrum disorders (i.e., schizophreniform disorder, schizophrenia, schizoaffective disorder). In this study, we examined whether habituation deficits identified in a cohort of early psychosis patients at baseline within a visual processing network—the hippocampus, FFA, and occipital pole—progressed over two years of illness. Across regions, patients habituated less than healthy control participants. In contrast to our hypothesis that deficits would progress, we found that habituation deficits in psychosis patients were maintained, but did not worsen, over 2 years. Furthermore, while healthy control participants habituated rapidly in all brain regions, patients showed a mixture of slowed or failed habituation. Habituation in the FFA was slower in patients than in healthy control participants, but importantly, was achieved in both groups. In contrast, habituation in the anterior hippocampus and occipital pole was not detected in patients, indicating abnormally sustained brain activity in these regions, or habituation failure. Together, these findings provide evidence for a persistent habituation deficit maintained throughout the early stage of schizophrenia.

Hippocampal deficits are among the most consistent and replicable findings in schizophrenia^63^, have been demonstrated at the earliest stages of illness^64,65^, and have been linked with inhibitory/excitatory signaling imbalance originating in the anterior CA1 region during early illness^63,65,66^. Although hippocampal deficits are thought to progress throughout illness^67,68^, the timeline of this progression remains unclear as few longitudinal studies of the hippocampus have been conducted^69^. We previously found anterior hippocampal habituation deficits in both early psychosis^17^ and chronic schizophrenia^16^ in response to repeated visual stimuli, suggesting habituation deficits persist into later stages of illness. Our current longitudinal findings provide further support for this notion, and preliminary evidence that hippocampal inhibitory deficits may not progress in the early years of illness. These results are also consistent with recent findings in a partially-overlapping cohort, which showed hippocampal volume is reduced in the early stage of psychosis^70^ but does not further decline over a 2-year follow-up period^71^. Together, these findings suggest that hippocampal deficits are stable in the first 2 years of schizophrenia. However, follow-up longitudinal studies at longer intervals will be necessary to determine how long this window of stability may last.

Similarly, our findings in the occipital pole and FFA suggest that habituation deficits do not progress over the early course of illness. While sensory gating deficits in schizophrenia are well described in the auditory system^72^, fewer studies have focused on the visual system. Those that have suggest visual deficits in schizophrenia are driven by impairment in the dorsal (“where”) visual stream, with relatively spared functioning in ventral (“what”) visual stream^72,73^. However, other findings support functional deficits in the ventral visual stream that may be related to stimulus discrimination in schizophrenia^74–76^. The occipital pole, a region including both the superior and inferior posterior occipital cortex, is involved in processing macular (central) vision^77^. The hippocampus receives visual sensory input via the ventral visual stream, including direct connections from the occipital and fusiform cortex with reciprocal feedback from the hippocampus to visual cortex, which is thought to support visual discrimination and learning. Thus, paradigms that require visual learning, such as habituation paradigms, may be well-suited to elicit inhibitory deficits in the ventral visual pathway and hippocampus.

Although molecular studies suggest habituation can result from complex local mechanisms at the synapse^78^, network-level inhibitory potentiation may also drive habituation response^79^. Although we detected habituation deficits across regions, the current findings cannot clarify whether habituation deficits originate in one or all identified regions. The hippocampus may be uniquely situated to influence responses across the visual network^80,81^. In schizophrenia, there is compelling evidence for interneuron dysfunction in the hippocampus^25^, and reciprocal connections with the visual system may result in continued feedback to visual cortices. An influential early model of habituation^82^ proposed that as a stimulus is repeated, feedback inhibition promotes a decrease in neural response and a memory trace^83^. Hippocampal activity exemplifies this learning response—the hippocampus responds strongly to novelty^84,85^ but rapidly suppresses responses to repeated information via local interneuron-mediated inhibitory feedback^86–89^. Additionally, hippocampal habituation has been associated with visual-stimulus memory in healthy individuals and in schizophrenia^16,17,90–92^. However, given evidence for deficient inhibition within the visual cortex^33,34^ in schizophrenia, it is notable that both the hippocampus and occipital pole showed a similar failure to habituate. Future studies should consider habituation designs that could inform directional influences across regions.

Our results should be interpreted within the context of study limitations. The goal of this study was to examine habituation longitudinally in an a priori-defined set of visual processing and memory regions that showed habituation deficits in a prior cohort analysis^17^. Whole-brain approaches may reveal additional habituation deficits in schizophrenia. We used a visual-stimulus repetition paradigm, which can be readily conducted in an fMRI environment. However, as auditory system sensory gating deficits are well described in schizophrenia at the cellular and molecular level^72^, the translational utility of habituation paradigms may be increased by examining habituation to auditory stimuli. We used a habituation model that allowed us to characterize habituation rate independent of novelty response within regions of interest. Approaches that can be used at the voxel-wise level may reveal greater regional specificity of habituation deficits. Habituation may be influenced by a variety of factors, including attention, anxiety, arousal, and saccadic suppression^20,59,93,94^. It is unlikely that our current findings are due to differences in attention, as target detection performance was high and did not differ between groups. However, future studies should consider measuring arousal, anxiety, and eye movements during the habituation session to account for potential influences.

In summary, we find evidence for persistent habituation deficits in schizophrenia. Over 2-year follow-up, early psychosis patients maintained slower or failed habituation across a set of regions including the hippocampus, occipital pole, and FFA. Although habituation is a ubiquitous and highly conserved process, individual differences have been reported in infancy^95,96^ and are hypothesized to fundamentally contribute to psychopathology^6,9,97,98^. Better understanding of translational mechanisms, such as habituation, are crucial in the exploration of novel interventions in schizophrenia^99^, and may be beneficial in understanding the mechanisms of onset and cognitive impairment associated with illness.

## Supplementary information

Supplementary Methods and Results
