# Supplementary material for: Stable habituation deficits in the early stage of psychosis: a 2-year follow-up study
Source: Transl Psychiatry. 2021 Jan 5;11:20. doi: 10.1038/s41398-020-01167-9 (PMC7791099; doi:10.1038/s41398-020-01167-9)
Supplement: Supplementary file 1 — Supplementary Methods and Results [file 41398_2020_1167_MOESM1_ESM.docx]

**SUPPLEMENTARY METHODS AND MATERIALS**

**Participants**

A group of 70 patients in the early stage of psychosis and 68 demographically-similar healthy control participants were recruited to complete a longitudinal neuroimaging study of habituation and followed for two years (Supplementary Figure 1). Of these, baseline data for 6 early psychosis patients and 1 healthy control participant were excluded due to: poor quality T1 image (1 patient); or failure to collect habituation scans (5 patients, 1 control). Six early psychosis patients and 12 healthy control participants were lost to follow-up but had an eligible diagnosis at baseline (schizophrenia or schizoaffective disorder). Follow-up data for an additional 5 early psychosis patients and 4 healthy control participants were excluded due to: ineligible follow-up data due to current substance use disorder (2 patients) or major depressive disorder between study visits (1 control); motion (1 patient, see *Quality control*); technical issues with stimulus delivery in the scanner (1 control); or failure to collect habituation scan (2 patients, 2 controls). This resulted in an early psychosis analysis group that included 53 baseline + follow-up scans, 11 baseline only scans, and 6 follow-up only scans. These were compared to a healthy control analysis group that included 51 baseline + follow-up scans, 16 baseline only scans, and 1 follow-up only scan (Supplementary Figure 2).

**Supplementary Figure 1.**


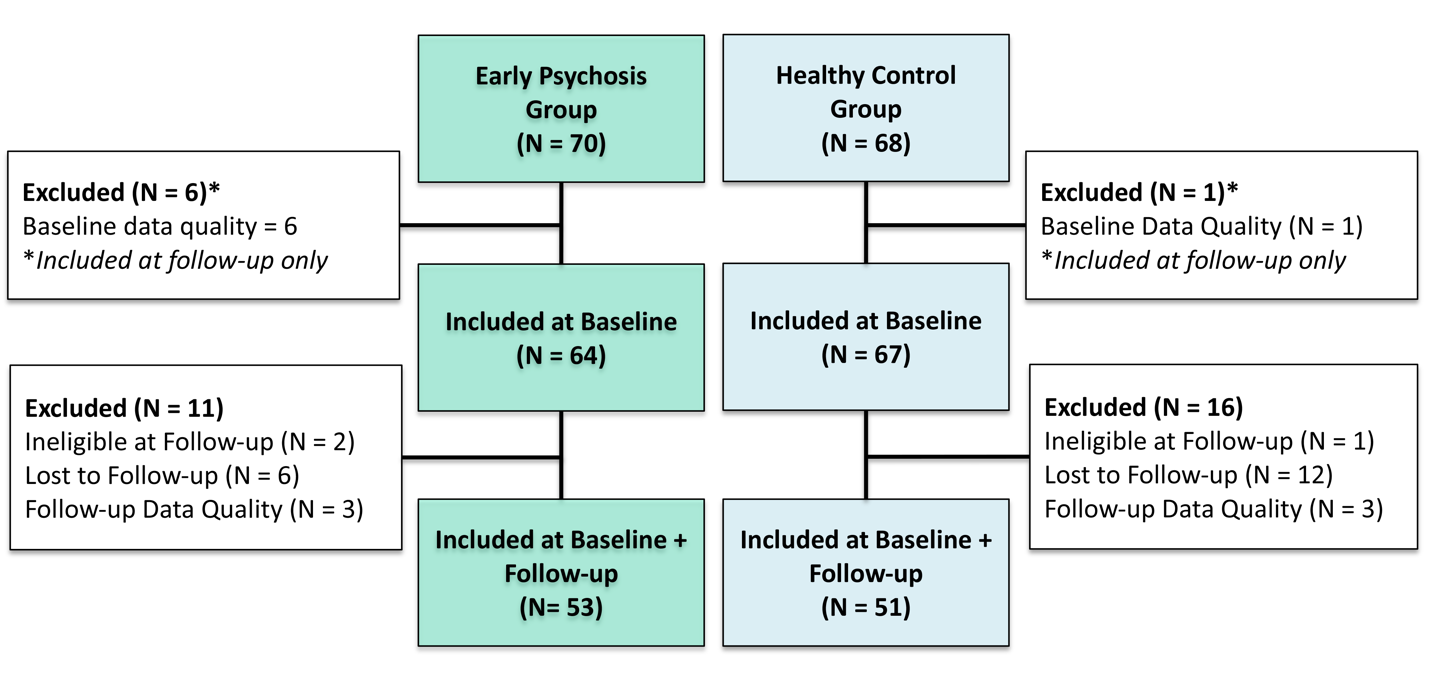


**Supplementary Figure 1.** Flow diagram of study participants.

**Supplementary Figure 2.**


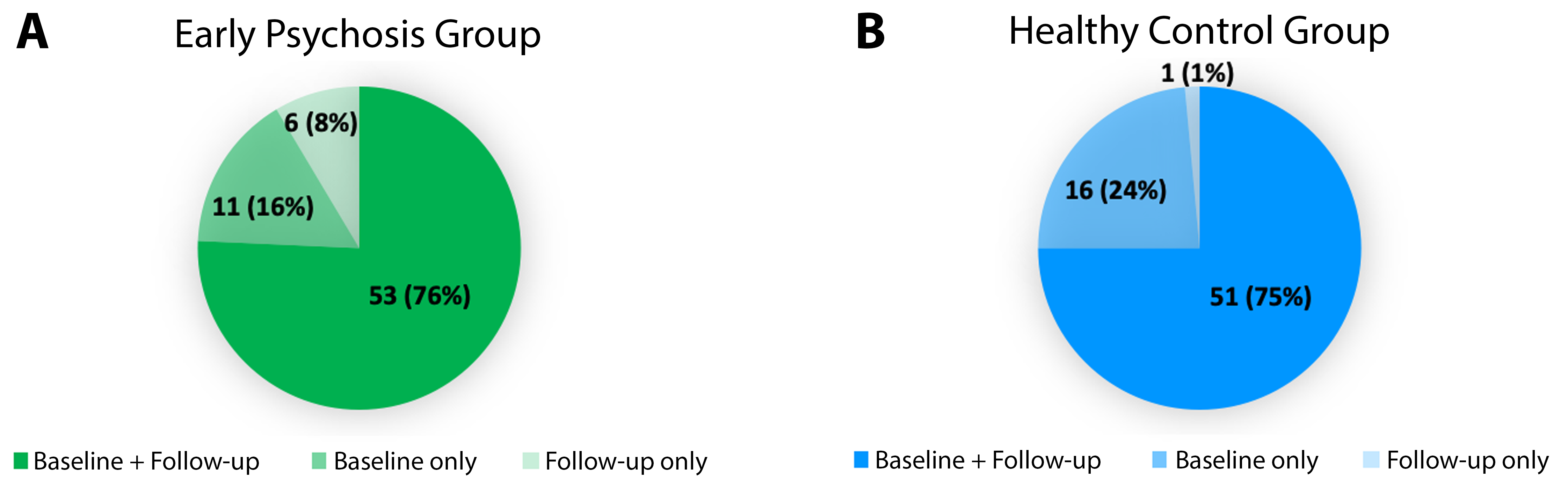


**Supplementary Figure 2.** Proportion of participants in the early psychosis group (A) and healthy control group (B) included at baseline and follow-up, baseline only, or follow-up only. The majority of participants were included at both study visits.

**Clinical Assessment and Measures**

Participants completed the Wechsler Test of Adult Reading (WTAR; Psychological Corporation, 2001) as a measure of premorbid IQ^1,2^. WTAR scores are reported for native English speakers (5 healthy control participants and 2 early psychosis patients were non-native English speakers). Patients were also assessed with the Positive and Negative Syndrome Scale (PANSS)^3,4^, the Hamilton Depression Rating Scale (HAM-D)^5^, and the Young Mania Rating Scale (YMRS)^6^. One patient did not have a YMRS score at baseline. Chlorpromazine (CPZ) equivalent doses were calculated according to Gardner *et al.*^7^ where possible. For patients taking lurasidone (n = 5; 2 at baseline, 3 at follow-up), CPZ equivalent was calculated according to Leucht *et al*.^8^. Twenty-four early stage psychosis patients were not receiving antipsychotics at the time of the study (3 at baseline, 16 at follow-up, 5 at neither visit). The onset of prodromal symptoms were determined using the Symptom Onset in Schizophrenia scale (SOS)^9^.

**Experimental paradigm**

*Stimuli.* During habituation runs, achromatic face or object images (345 x 401 pixels, 72 dpi) were presented at the center of the screen for 500 ms followed by a 500 ms blank screen. All faces had a neutral expression, and were taken from standard stimulus sets^10–13^. Images of common, neutral objects (e.g. an umbrella, a vase, a lamp) were obtained from internet photo databases.

*Repetition task design.* The repetition task is shown in Supplementary Figure 3. During each face run the same face (1 run male, 1 run female) or object (table, box of tissues) was repeated 120 times. Face runs were always presented first to match the design of previous studies^14^. Each run began with a 10 second (s) fixation screen and ended with 20 s of fixation. Participants viewed a blank screen for approximately 20 s between stimulus presentation runs. To assess attention, small versions of the face or object images (25% of original size) were presented on 10% of trials, and participants were asked to respond to small images with a button press. Five participants had low response rates (< 10% of trials; healthy control, n = 3 at baseline; early psychosis, n = 1 at baseline, n = 1 at follow-up). Habituation rates for these participants did not differ from the rest of the group (*p*s ≥ 0.71) and findings were not affected by exclusion of these participants; therefore, the participants were included in the analysis.

**Supplementary Figure 3.**


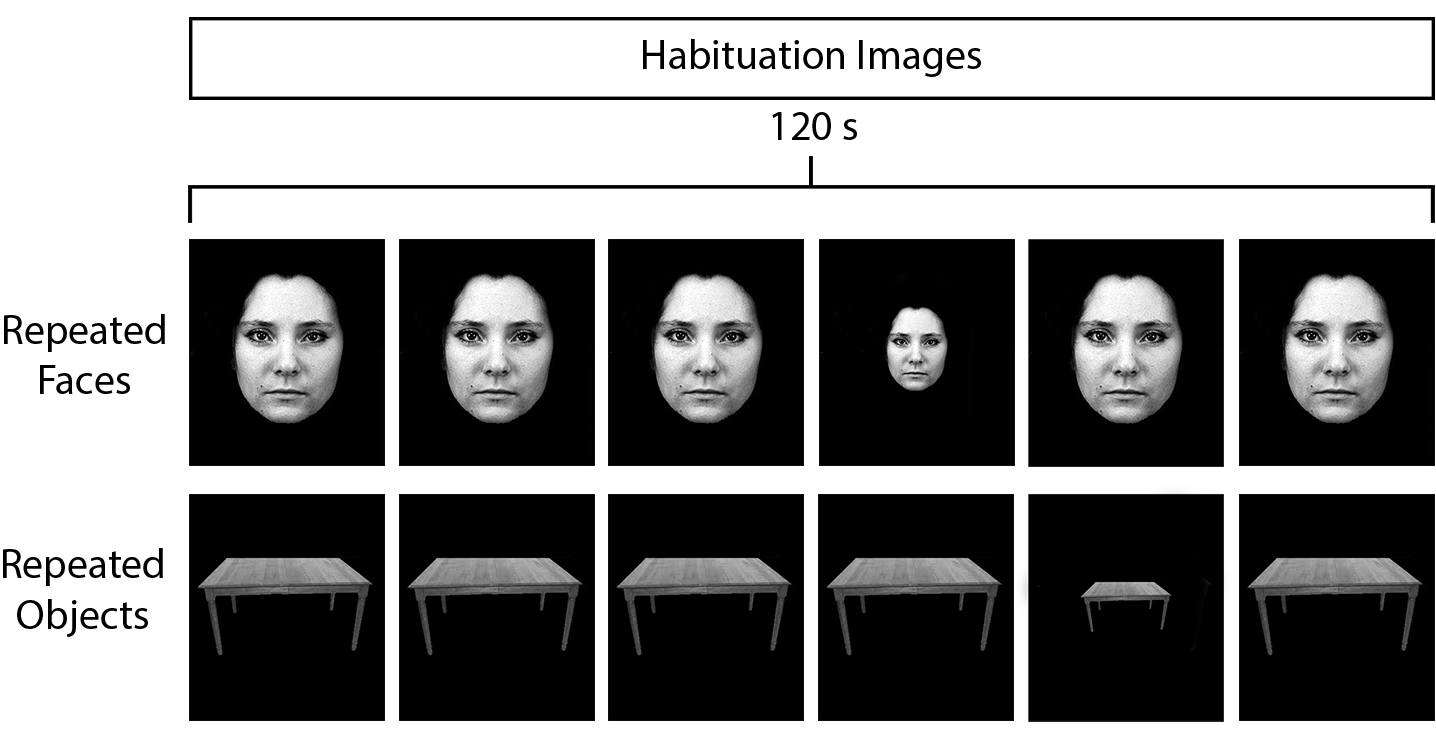


**Supplementary Figure 3.** Repetition task. Participants viewed repeated images of faces (2 consecutive runs) and objects (2 consecutive runs). Participants pressed a button to detect small images (10% of trials), which were excluded from the habituation analysis.

**Imaging data acquisition and processing**

For each participant, a high-resolution T1-weighted fast field echo (FFE) structural scan was acquired with the following parameters: 170 sagittal slices; matrix = 256 x 256; 1 mm isotropic resolution; TR = 8.0 ms; TE = 3.7 ms. Following the structural scan, four functional FFE echo planar images (EPI) were acquired using a sequence optimized to reduce signal loss in the temporal lobe and ventral forebrain: 2 s TR; 28 ms TE; 90° flip angle; EPI factor = 43; 240 mm FOV; 3 x 3 mm in-plane resolution using an 80 x 80 matrix; and higher order shimming to limit susceptibility artifacts. Each volume contained 38 3.2 mm (0 gap) axial oblique slices (tilted 15° anterior higher than posterior relative to the intercommissural plane), which provided whole-brain coverage. Each functional scan was 2.5 minutes in length (75 volumes).

**Data analysis**

*Regions of interest (ROIs).* Region of interest masks are shown in Supplementary Figure 2. Because the precise location of the fusiform face area (FFA) cannot be anatomically defined and differs across individuals, it is necessary to identify the FFA using a functional task. Individual FFA ROIs were calculated for each participant using a standard FFA localizer task^15^. We imposed several constraints on localizer data to ensure that activations were located within the FFA: first, activations were constrained within the boundaries of the fusiform gyrus (AAL; WFU pickatlas); second, to minimize the chance of spurious activations being included in the mask, a cluster-based threshold was applied to activations—a minimum cluster size of 8 voxels (*p* = .005) provided a cluster-corrected α = .05; finally, statistical thresholds were adjusted for each participant to constrain activations to a cluster size representative of FFA volume—a maximum cluster size of 37 voxels (999 mm^3^) in the right fusiform and 19 voxels (513 mm^3^) in the left fusiform. Maximum cluster sizes were based on a review of published studies reporting FFA volumes^16^. Individual ROIs were maintained for analysis, as previous studies have shown that this approach is stronger, relative to a group overlap ROI, in selectively analyzing face processing signal^17^.

*Habituation slopes.* Neural habituation slopes were modeled for each participant using the regression

$$Y=bX+a$$

where the mean ROI response (Y) is predicted by the log-transformed face presentation number (X). Face presentations were natural log-transformed. The natural log transform linearizes the habituation curve, which is steepest during early face repetitions, enabling linear regression analysis. We then calculated b' for each participant as

$$b'=b-c(a- \bar{a})$$

where b is the participant’s regression slope, c is the mean regression parameter estimate (time) of the sample, and a is the initial amplitude estimate (intercept).

**SUPPLEMENTARY RESULTS**

**Motion**

Motion was low and similar between groups (Supplementary Table 1).

**Supplementary Table 1. Motion.**

|  | | Healthy control | | Early Psychosis | | Healthy control vs. Early psychosis | |
| --- | --- | --- | --- | --- | --- | --- | --- |
|  | | Mean ± SD | Max | Mean ± SD | Max | *F* | *p* |
| Baseline | |  |  |  |  |  |  |
|  | Mean translation (mm) | 0.10 ± 0.16 | 1.22 | 0.08 ± 0.07 | 0.40 | 0.72 | 0.40 |
|  | Mean rotation (degrees) | 0.10 ± 0.19 | 1.29 | 0.09 ± 0.13 | 0.82 | 0.00 | 0.98 |
|  | Median relative displacement (mm) | 0.20 ± 0.14 | 0.72 | 0.18 ± 0.10 | 0.52 | 2.04 | 0.16 |
| 2 year follow-up | |  |  |  |  |  |  |
|  | Mean translation (mm) | 0.08 ± 0.08 | 0.53 | 0.09 ± 0.13 | 0.88 | 0.57 | 0.45 |
|  | Mean rotation (degrees) | 0.07 ± 0.08 | 0.49 | 0.07 ± 0.07 | 0.28 | 0.28 | 0.60 |
|  | Median relative displacement (mm) | 0.20 ± 0.16 | 1.00 | 0.19 ± 0.33 | 2.35 | 1.34 | 0.25 |

**Target detection**

Response rates during small image presentations were high and did not differ between groups (baseline: control mean = 93%, SD = 21%; patient mean = 94%, SD = 15%; follow-up: control mean = 95%, SD = 8%; patient mean = 93%, SD = 17%; *p*s ≥ 0.36) or across study visits (*p* = 0.79). Reaction times were also similar between groups (baseline: control mean = 462 ms, SD = 58 ms; patient mean = 466 ms, SD = 65 ms; follow-up: control mean = 508 ms, SD = 60 ms; patient mean = 514 ms, SD = 73 ms; *p*s > 0.63). Across participants, baseline reaction times were faster than follow-up reaction times (*t*_96_ = -7.50, *p* < 0.001). Habituation rates were not correlated with behavioral response to small faces (baseline, *p*s ≥ 0.36; follow-up, *p*s ≥ 0.14).

**Novelty response**

Novelty responses in each region were similar between groups (*p*s ≥ 0.76) and did not differ over time (*p*s ≥ 0.52; Supplementary Table 2). We conducted planned one-sample *t*-tests to test whether novelty responses were significantly greater than zero. All regions showed a greater than baseline response to novel objects (Supplementary Table 3).

**Supplementary Table 2. Novelty response by group.**

| Brain Region | | Healthy Control | Early Psychosis | Main Effect of Group | | | Main Effect of Time | | |
| --- | --- | --- | --- | --- | --- | --- | --- | --- | --- |
|  |  | M ± SD | M ± SD | *F* | *df* | *p* | *F* | *df* | *p* |
|  | Anterior hippocampus | 0.15 ± 0.7 | 0.11 ± 0.6 | 0.04 | 1,136 | 0.84 | 0.42 | 1,102 | 0.52 |
|  | Occipital pole | 0.16 ± 0.5 | 0.13 ± 0.6 | 0.07 | 1,136 | 0.80 | 0.05 | 1,102 | 0.82 |
|  | FFA | 0.62 ± 0.5 | 0.64 ± 0.6 | 0.09 | 1,136 | 0.76 | 0.25 | 1,93 | 0.62 |

Note: Mean values ± standard deviations are shown for each group. M, mean; SD, standard deviation; FFA, fusiform face area.

**Supplementary Table 3. Novelty response across participants.**

| Brain region | Novelty response | | |
| --- | --- | --- | --- |
|  | Mean ± SD | *t* (h0=0) | *p* |
| Anterior hippocampus | 0.10 ± 0.5 | 2.44 | 0.008* |
| Occipital pole | 0.12 ± 0.4 | 3.49 | < 0.001* |
| FFA | 0.61 ± 0.5 | 14.07 | < 0.001* |

Note: Mean values ± standard deviations are shown for each group. M, mean; SD, standard deviation; FFA, fusiform face area. Asterisk (*) denotes significant *p*-values (*p* < 0.05).

**Uncorrected habituation slope**

We conducted a secondary analysis examining habituation slopes uncorrected for novelty response (*b*). Group results were broadly similar to the primary habituation (*b*′) analysis, with early psychosis patients habituating approximately 10-15% slower than healthy control participants across regions (Supplementary Table 4). The main difference was that group effects in the FFA were not significant in the uncorrected slopes analysis, with the effect size dropping from moderate (*b*′ ES = 0.58) to small (*b* ES = 0.17). No regions showed a main effect of time (*p*s ≥ 0.19) or a group by time interaction (*p*s ≥ 0.29).

**Supplementary Table 4. Uncorrected habituation slope.**

| Brain Region | | Healthy Control | Early Psychosis | Main Effect of Group | | | | Main Effect of Time | | |
| --- | --- | --- | --- | --- | --- | --- | --- | --- | --- | --- |
|  |  | M ± SD | M ± SD | *F* | *df* | ES | *p* | *F* | *df* | *p* |
|  | Anterior hippocampus | -0.18 ± 0.5 | -0.03 ± 0.5 | 4.03 | 1,136 | 0.30 | 0.04* | 0.80 | 1,102 | 0.37 |
|  | Occipital pole | -0.14 ± 0.4 | 0.07 ± 0.5 | 6.92 | 1,136 | 0.42 | 0.009* | 0.01 | 1,102 | 0.94 |
|  | FFA | -0.43 ± 0.5 | -0.34 ± 0.5 | 2.01 | 1,136 | 0.17 | 0.16 | 1.75 | 1,93 | 0.19 |

Note: Mean values ± standard deviations are shown for each group. M, mean; SD, standard deviation; ES = effect size; FFA, fusiform face area. Asterisk (*) denotes significant *p*-values (*p* < 0.05).

**Correlates of habituation**

We tested for effects of potential moderators of habituation, including medication dose (chlorpromazine equivalent), psychosis symptoms (PANSS; positive, negative, and general subscale), duration of illness, and mood (YMRS, HAMD). Spearman correlations were corrected for multiple comparisons across measures within group, time, and region (FWE-adjusted *p* ≤ 0.05). There was a small negative correlation between negative symptoms and habituation in the anterior hippocampus at follow-up (*r* = -0.38, FWE-adjusted *p* = 0.03). No other clinical or cognitive measures were correlated with habituation rate (Supplementary Table 5).

**Supplementary Table 5. Habituation rate by cognition and clinical symptoms.**

| Baseline | | Anterior hippocampus | | Occipital pole | | FFA | |
| --- | --- | --- | --- | --- | --- | --- | --- |
|  |  | *r* | FWE-adjusted *p* | *r* | FWE-adjusted *p* | *r* | FWE-adjusted *p* |
| Healthy control | |  |  |  |  |  |  |
|  | General cognition (SCIP) | -0.11 | 0.66 | -0.18 | 0.29 | -0.15 | 0.58 |
|  | IQ (WTAR) | -0.13 | 0.66 | -0.22 | 0.26 | -0.07 | 0.58 |
| Early Psychosis | |  |  |  |  |  |  |
|  | General cognition (SCIP) | -0.22 | 0.80 | 0.01 | 1.00 | -0.27 | 0.29 |
|  | IQ (WTAR) | -0.20 | 1.00 | -0.08 | 1.00 | -0.18 | 1.00 |
|  | PANSS-Total | 0.01 | 1.00 | -0.20 | 1.00 | -0.03 | 1.00 |
|  | PANSS-Positive | -0.04 | 1.00 | -0.11 | 1.00 | 0.09 | 1.00 |
|  | PANSS-Negative | -0.01 | 1.00 | -0.17 | 1.00 | -0.02 | 1.00 |
|  | PANSS-General | 0.06 | 1.00 | -0.15 | 1.00 | -0.08 | 1.00 |
|  | Duration of illness | 0.03 | 1.00 | 0.03 | 1.00 | -0.13 | 1.00 |
|  | CPZ dose (mg) | 0.13 | 1.00 | -0.24 | 0.57 | 0.07 | 1.00 |
|  | YMRS | -0.18 | 1.00 | -0.03 | 1.00 | -0.07 | 1.00 |
|  | HAMD | -0.03 | 1.00 | -0.04 | 1.00 | -0.10 | 1.00 |
| 2 year follow-up | | Anterior hippocampus | | Occipital pole | | FFA | |
|  |  | *r* | FWE-adjusted *p* | *r* | FWE-adjusted *p* | *r* | FWE-adjusted *p* |
| Healthy control | |  |  |  |  |  |  |
|  | General cognition (SCIP) | 0.03 | 1.00 | -0.07 | 0.64 | -0.18 | 0.40 |
|  | IQ (WTAR) | 0.00 | 1.00 | -0.15 | 0.58 | -0.09 | 0.54 |
| Early psychosis | |  |  |  |  |  |  |
|  | General cognition (SCIP) | 0.15 | 1.00 | 0.08 | 1.00 | -0.05 | 1.00 |
|  | IQ (WTAR) | 0.09 | 1.00 | -0.13 | 1.00 | 0.12 | 1.00 |
|  | PANSS-Total | -0.29 | 0.25 | -0.16 | 1.00 | -0.08 | 1.00 |
|  | PANSS-Positive | -0.03 | 1.00 | -0.14 | 1.00 | 0.13 | 1.00 |
|  | PANSS-Negative | -0.38 | 0.03* | -0.18 | 1.00 | -0.10 | 1.00 |
|  | PANSS-General | -0.24 | 0.55 | -0.08 | 1.00 | -0.07 | 1.00 |
|  | Duration of illness | -0.10 | 1.00 | 0.02 | 1.00 | -0.02 | 1.00 |
|  | CPZ dose (mg) | 0.04 | 1.00 | -0.05 | 1.00 | 0.06 | 1.00 |
|  | YMRS | -0.07 | 1.00 | -0.06 | 1.00 | 0.12 | 1.00 |
|  | HAMD | -0.15 | 1.00 | -0.31 | 0.19 | -0.03 | 1.00 |

Note: FFA, fusiform face area. Asterisk (*) denotes significant *p*-values (FWE-adjusted *p* ≤ 0.05).

**REFERENCES**

1 Dykiert D, Deary IJ. Retrospective validation of WTAR and NART scores as estimators of prior cognitive ability using the Lothian Birth Cohort 1936. *Psychol Assess* 2013; **25**: 1361–1366.

2 Dalby JT, Williams R. Preserved reading and spelling ability in psychotic disorders. *Psychol Med* 1986; **16**: 171–5.

3 Kay SR *et al.* The positive and negative syndrome scale (PANSS) for schizophrenia. *Schizophr Bull* 1987; **13**: 261–76.

4 Kay SR, Opler LA, Lindenmayer JP. Reliability and validity of the positive and negative syndrome scale for schizophrenics. *Psychiatry Res* 1988; **23**: 99–110.

5 Hamilton M. A rating scale for depression. *J Neurol Neurosurg Psychiatry* 1960; **23**: 56–62.

6 Young RC, Biggs JT, Ziegler VE, Meyer DA. A rating scale for mania: reliability, validity and sensitivity. *Br J Psychiatry* 1978; **133**: 429–435.

7 Gardner DM, Murphy AL, O’Donnell H, Centorrino F, Baldessarini RJ. International consensus study of antipsychotic dosing. *Am J Psychiatry* 2010; **167**: 686–93.

8 Leucht S *et al.* Dose equivalents for second-generation antipsychotics: The minimum effective dose method. *Schizophr Bull* 2014; **40**: 314–326.

9 Perkins DOO *et al.* Characterizing and dating the onset of symptoms in psychotic illness: the Symptom Onset in Schizophrenia (SOS) inventory. *Schizophr Res* 2000; **44**: 1–10.

10 Gur RCRECE *et al.* Computerized neurocognitive scanning: I. Methodology and validation in healthy people. *Neuropsychopharmacology* 2001; **25**: 766–76.

11 Lundqvist D, Flykt A, Ohman A. The Karolinska Directed Emotional Faces - KDEF. 1998.

12 Minear M, Park DC. A lifespan database of adult facial stimuli. *Behav Res Methods Instrum Comput* 2004; **36**: 630–3.

13 Tottenham N *et al.* The NimStim set of facial expressions: Judgments from untrained research participants. *Psychiatry Res* 2009; **168**: 242–249.

14 Williams LE, Blackford JU, Luksik A, Gauthier I, Heckers S. Reduced habituation in patients with schizophrenia. *Schizophr Res* 2013; **151**: 124–132.

15 Wong YK, Gauthier I. A multimodal neural network recruited by expertise with musical notation. *J Cogn Neurosci* 2010; **22**: 695–713.

16 Berman MG *et al.* Evaluating functional localizers: The case of the FFA. *Neuroimage* 2010; **50**: 56–71.

17 Saxe R, Brett M, Kanwisher N. Divide and conquer: A defense of functional localizers. *Neuroimage* 2006; **30**: 1088–1096.
